# Directly Grown Multiwall Carbon Nanotube and Hydrothermal MnO_2_ Composite for High-Performance Supercapacitor Electrodes

**DOI:** 10.3390/nano9050703

**Published:** 2019-05-06

**Authors:** Li Li, Lihui Chen, Weijin Qian, Fei Xie, Changkun Dong

**Affiliations:** Institute of Micro-nano Structures & Optoelectronics, Wenzhou University, Wenzhou 325035, China; lili18767702665@gmail.com (L.L.); chenlihui19880416@gmail.com (L.C.); weijinqian@wzu.edu.cn (W.Q.); xiefei600@gmail.com (F.X.)

**Keywords:** carbon nanotube, supercapacitor, chemical vapor deposition, manganese dioxide, hydrothermal method

## Abstract

MnO_2_–MWNT–Ni foam supercapacitor electrodes were developed based on directly grown multiwalled carbon nanotubes (MWNTs) and hydrothermal MnO_2_ nanostructures on Ni foam substrates. The electrodes demonstrated excellent electrochemical and battery properties. The charge transfer resistance dropped 88.8% compared with the electrode without MWNTs. A high specific capacitance of 1350.42 F·g^−1^ was reached at the current density of 6.5 A·g^−1^. The electrode exhibited a superior rate capability with 92.5% retention in 25,000 cycles. Direct MWNT growth benefits the supercapacitor application for low charge transfer resistance and strong MWNT–current collector binding.

## 1. Introduction

The supercapacitor, mainly the electrical double-layer capacitor (EDLC) and the pseudocapacitor, is of interest for quick charging/discharging capacity, high power density, and long cycle life [1,2]. There are three main types of electrodes, namely, carbon nanomaterials, metal oxides/hydroxides, and polymers [3,4,5,6]. Supercapacitor devices constructed upon carbon nanomaterials, including carbon nanotube (CNT) and graphene, have been widely investigated and have shown remarkable electrochemical properties [7,8,9,10]. Wei et al. prepared a 3D nanostructure by growing carbon nanotubes between graphene layers, which exhibited good electrochemical properties with a specific capacitance of 385 F·g^−1^ [11]. In pseudocapacitor applications, various metal oxides, for example cobalt tetraoxide [10,12], nickel oxide [13], and manganese dioxide [14,15], have been employed for excellent multiple oxidation states during charging/discharging. Having high specific capacitance, excellent cycle stability, and an environmentally friendly nature, MnO_2_ is typically employed as the active material to further improve the energy efficiency of CNT-based electrodes, and CNT–MnO_2_ composite electrodes have shown great advantages in pseudocapacitors [16,17,18,19,20]. Hu et al. synthesized the MnO_2_–CNT textile composite, and the specific capacitance retention of 60% was reached after 10,000 cycles [20]. Typically, the MnO_2_–CNT composite electrode is made from either the addition of CNT powder [21] or by growing CNTs on catalyst film [22]. The current collector–carbon interface is considered the key source to generate electronic impedance [23]. However, the development of supercapacitor electrodes by growing CNTs directly on Ni foam, which has the advantage of lowering the substrate–CNT contact resistance, has not attracted broad attention and is less reported.

In this study, we fabricated a MnO_2_–MWNT–Ni foam composite electrode by the hydrothermal production of MnO_2_ after synthesizing multiwalled carbon nanotubes (MWNTs) directly on a catalytic nickel foam surface by chemical vapor deposition (CVD). The composite electrode showed good electrochemical properties with a specific capacitance of 1350.42 F·g^−1^ at 6.5 A·g ^−1^, and 93.9% retention was reached after 4600 cycles under the areal density of 0.775 mg·cm^−2^. The MnO_2_–MWNT–Ni foam composite exhibited a unique conductive network from direct and strong contacts between the MWNTs and the current collector, significantly enhancing the structural, electronic, and electrochemical properties. The approach of direct MWNT growth is promising to enable the development of high capacitance pseudocapacitors with high energy densities and a long cycle life.

## 2. Materials and Methods

### 2.1. Synthesis of the MnO_2_–MWNT–Ni Foam Composite Electrode

**Figure 1:**
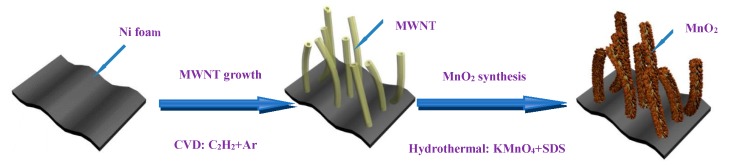
Production process of MnO_2_–MWNT–Ni foam composite. MWNT: multiwalled carbon nanotube.

There were three main processes in the production of the MnO_2_–MWNT–Ni foam composite electrode. Firstly, MWNTs were synthesized on the Ni foam directly at 680 °C with a 20/200 sccm flow rate of acetylene/argon [24,25]. Secondly, the MnO_2_–MWNT–Ni foam composite sample was prepared from the reaction of KMnO_4_ (4 × 10^−3^ g·mL^−1^) with sodium dodecyl sulfate (SDS, 2 × 10^−3^ g·mL^−1^) in a reaction kettle under 120 °C for 10 h and dried in an oven at 50 °C. In the third step, the composite material was reinforced with polytetrafluoroethylene (PTFE) and dried at 50 °C for 1 h. Two pieces of as-formed electrodes loaded with the hybrid were then pressed together at 30 MPa for 30 s. The total mass of the electrode was 0.2872 g, with the active material having a mass of 0.0062 g. The core processes of MWNT growth and MnO_2_ synthesis are illustrated in Figure 1.

### 2.2. Characterization

The MnO_2_–MWNT–Ni foam structure was analyzed by Scanning Electron Microscope (SEM, JEOL 6700 F, Tokyo, Japan), high-resolution Transmission Electron Microscope (TEM, JEM-2100F, JEOL, Tokyo, Japan), X-Ray Diffraction (XRD, Bruker D8, Cu Kα radiation from 10 to 80 angles, Bruker AXS Inc., Karlsruhe, Germany), and Raman spectroscopy (Renishaw Invia Raman Microscope, with an excitation wavelength of 633 nm, Renishaw plc., Wotton under Edge, Gloucestershire, UK).

### 2.3. Electrochemical Measurements

Cyclic voltammetrys (CVs), electrochemical impedance spectroscopy (EIS), and galvanostatic charging–discharging (GCD) properties were investigated using an electrochemical workstation (Zennium, Zahner Instruments Inc., Kronach, Germany) with the three-electrode system, while the cyclic GCD test was performed by a battery testing system (LAND, RAMBO, Wuhan, China). The Hg/HgO electrode, Pt sheet, and as-prepared sample were used as the reference, counter, and working electrodes, respectively, in 6 M KOH solution. EIS measurements were scanned (10^−2^–10^6^ Hz) at the equilibrium conditions.

## 3. Results and Discussion

### 3.1. Structure Characterization

MnO_2_ nanoflakes were synthesized on Ni foam and MWNT–Ni foam substrates through the self-limiting reaction between KMnO_4_ and SDS using the wet chemical hydrothermal process [26]. The reaction can be described based on the following equation: [27,28]

(1) $$2{\mathrm{KMnO}}_{4}\to K_{2}{\mathrm{MnO}}_{4}+{\mathrm{MnO}}_{2}+O_{2}↑$$

**Figure 2:**
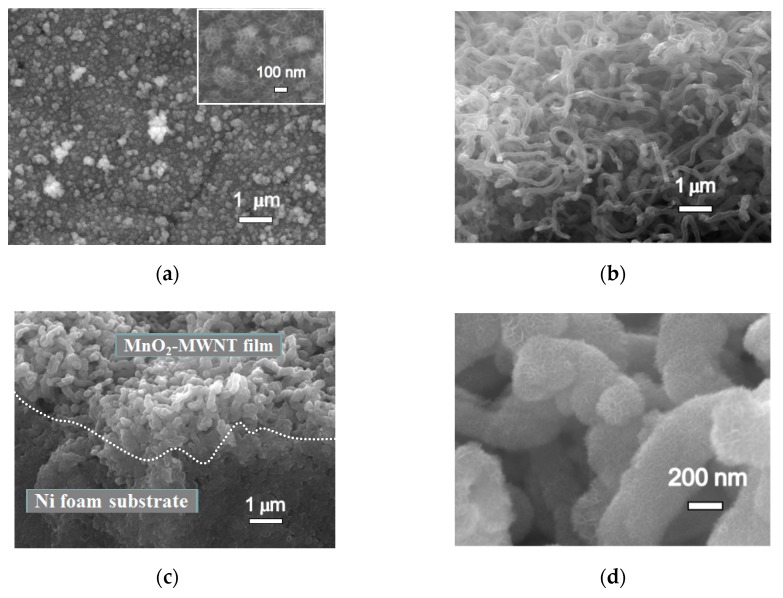

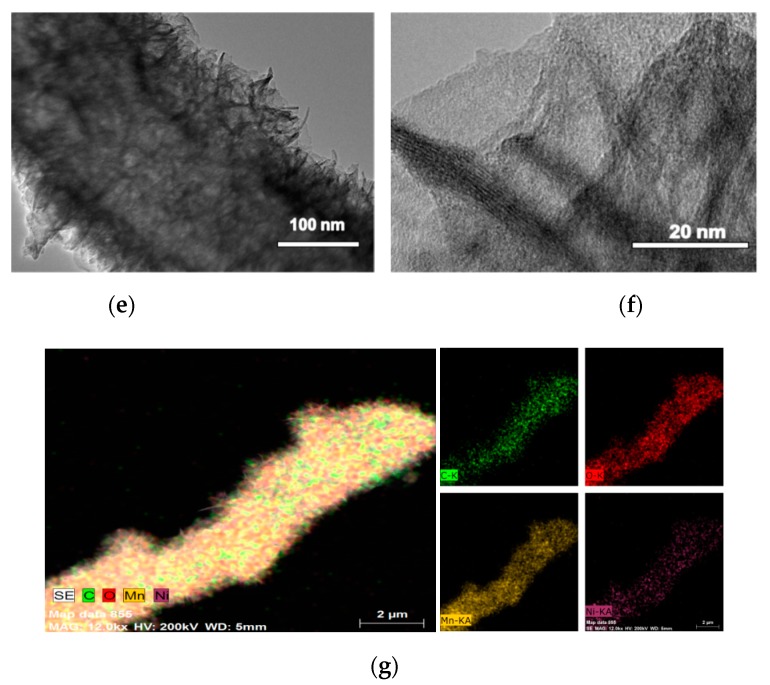
Electron micrographs of the MnO_2_–Ni foam and MnO_2_–MWNT–Ni foam composites, and EDS mapping of the MnO_2_–MWNT–Ni foam composite. (**a**–**d**) SEM images and (**e**–**f**) TEM images: (**a**) MnO_2_ synthesized on Ni Foam, (**b**) MWNTs grown directly on a Ni foam substrate, (**c**) cross-section of the MnO_2_–MWNT–Ni foam composite, (**d**,**e**) MnO_2_ synthesized uniformly on MWNTs with diameters of about 300 nm, and (**f**) MnO_2_ nanoflakes of less than 10 layers. (**g**) EDS mapping of the MnO_2_–MWNT–Ni foam composite.

The unique forest-like structure of a MWNT sets up a conductive network which greatly improves the electronic conductivity of the MnO_2_–MWNT–Ni foam [29]. Figure 2a demonstrates SEM images of the MnO_2_ nanoflake film on the Ni foam surface. Figure 2b shows randomly oriented MWNTs with diameters of ~70 nm and lengths of up to 20 μm. Figure 2c illustrates the cross-section of the MnO_2_–MWNT–Ni foam composite. There is no obvious boundary between the MnO_2_–MWNT film and the substrate, indicating excellent binding properties which benefited from the synthesis of MWNTs with the Ni foam directly. The MnO_2_ nanoflake structures grew uniformly on the MWNTs surfaces with an overall radius of around 150 nm and the nanoflake thickness of about 100 nm (Figure 2d,e). Such nanoflakes are normally less than 10 layers, with the interplanar distance in the 0.47~0.64 nm range (Figure 2f). The MnO_2_ nanoflakes benefit from the movement of electrolyte ions [30], greatly enhancing the specific surface area of the nanocomposite. Energy Dispersive Spectrometer (EDS mapping for the MnO_2_–MWNT–Ni foam composite uniformly shows the anticipated C, O, Mn, and Ni signals (Figure 2g).

**Figure 3:**
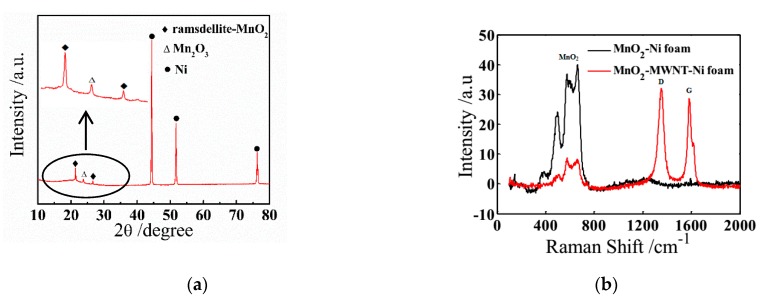
(**a**) XRD patterns of the MnO_2_–MWNT–Ni foam composites. (**b**) Raman spectra of the MnO_2_–Ni foam and MnO_2_–MWNT–Ni foam composites.

As for the XRD spectra of the MnO_2_–MWNT–Ni foam composite shown in Figure 3a, the characteristic diffraction peaks for the ramsdellite–MnO_2_ (JCPDS 42-1316) at 21.4° and 26.6° can be identified, and the weak peak at 23.7° is considered as the characteristic peak for (012) Mn_2_O_3_ (JCPDS 33-0900). The existence of Mn_2_O_3_ might be attributed to the treatment of the sample with ethanol [31]. The high specific surface and superior electrochemical stability of Mn_2_O_3_ benefit the enhancement of the specific capacitance [32,33]. The Raman spectra of the MnO_2_–Ni and MnO_2_–MWNT–Ni foams are shown in Figure 3b. The characteristic D peak at 1350 cm^−1^ and G peak at 1580 cm^−1^ are associated with MWNTs. The three peaks at 509.2, 577.3, and 659.5 cm^−1^ correspond to three characteristic vibrations of MnO_2_ compounds [34,35].

### 3.2. Electrochemical Measurements

**Figure 4:**
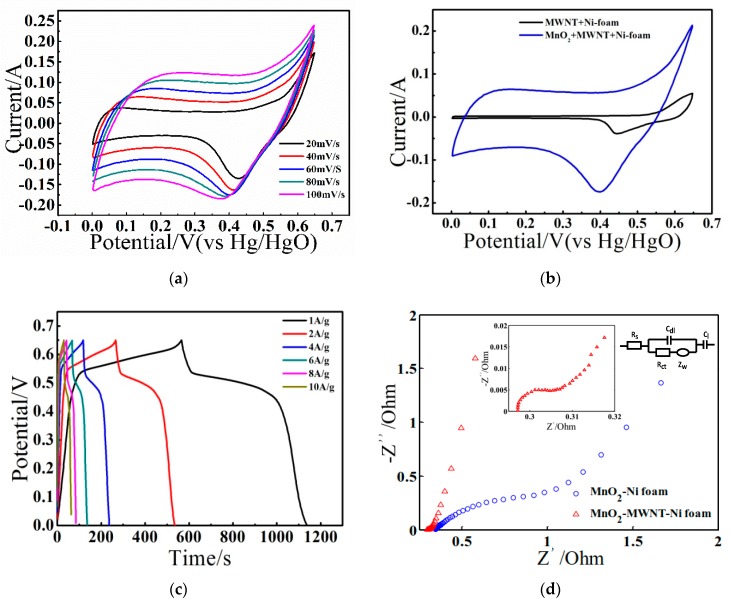
(**a**) Cyclic voltammetrys (CVs) of the MnO_2_–MWNT–Ni foam electrode at different scan rates. (**b**) CV comparison of the CNT–Ni foam electrode and the MnO_2_–MWNT–Ni foam electrode at 50 mV/s. (**c**) Charge/discharge curves of the MnO_2_–MWNT–Ni foam at different current densities in the electrochemical workstation. (**d**) Electrochemical impedance spectroscopy (EIS) curves of the MnO_2_–Ni foam and MnO_2_–MWNT–Ni foam electrodes tested in 6 M KOH.

Figure 4a shows the CV curves within 0–0.65 V, which exhibit clear redox peaks and gradually form a rectangular shape with the increase of the scan rate (more information regarding CVs between four types of electrodes can be found in supplementary materials Figure S1). Generally, the MnO_2_ electrode presents a double-layer capacitance with the CV curve of a square-like shape in K_2_SO_4_ or Na_2_SO_4_ aqueous solution [36]. However, the MnO_2_–MWNT–Ni foam electrode exhibited a pair of peaks in the KOH electrolyte, which are related to the insertion and extraction of hydrated K^+^ between the MnO_2_ and layers [37,38,39]. On the other hand, the NiO component on the Ni foam surface also played key role. As shown in SI-1, the Ni foam electrode exhibited such a pair of peaks, indicating the faradic pseudocapacitance of NiO [40]. Hence, the MnO_2_–MWNT–Ni foam electrode demonstrated both double-layer capacitance and faradic pseudocapacitance behaviors. However, due to the weakening of the NiO peaks after the MWNT growth and the overlap with the redox peaks from K^+^ intercalaction, it would be difficult to discriminate the two processes from the CV curve of the composite electrode. According to Figure 4c, the GCD curves show potential plateaus, which are attributed to the quasi-reversible faradic pseudocapacitance of NiO [41].

Figure 4d illustrates the EIS Nyquist plots of the MnO_2_–Ni foam and MnO_2_–MWNT–Ni foam electrodes. The impedance information, including the internal resistance (R_s_), the diffusion resistance (Z_w_) from the electrolyte, the charge transfer resistance (R_ct_), the double-layer capacitance (C_dl_), and the pseudocapacitance (C_l_), can be derived from the EIS data [42,43,44], as shown in the equivalent circuit. R_s_ depends mainly on the electrolyte ionic resistance and the contact resistance between the MWNTs and the Ni foam substrate [45]. The semicircle diameter is associated with R_ct_. R_ct_ is 0.411 Ω and 0.046 Ω, respectively, for the MnO_2_–Ni foam and the MnO_2_–MWNT–Ni foam electrodes, a significant reduction due to the participation of MWNTs. The improvement in electrical conductivity and the reduction of the charge transfer resistances with the addition of the MWNT layer are attributed to three aspects, namely, the superior electric properties of the MWNTs, the low charge transfer resistance through the MWNTs, and the reduction of contact resistances between the MWNTs and the current collector (Ni foam).

The MnO_2_–MWNT–Ni foam electrode demonstrated excellent battery properties, including high specific capacitance, excellent charging/discharging stability, as well as long cycle life. The specific capacitance is derived from the galvanostatic charging/discharging curve following the equation

(2) $$C_{m}=\;\frac{i_{m}\int Vdt}{\Delta V^{2}}$$

where *i_m_* is the current density (A/g), ∫Vdt is the integral current area, Δ*V* is the difference between the incipient discharge voltage and the final discharge voltage (V) [46]. In our experiment, the specific capacitance was calculated based on the total active material (0.0062 g) attached to the MWNT–Ni foam substrate measuring 2 cm × 2 cm. The energy density (E, Wh/kg) and power density (P, W/kg) can be calculated from galvanostatic tests by the following equations: E = [$C_{m}{\left(\Delta V\right)}^{2}$]/2 and P = E/Δt. The MnO2–MWNT–Ni foam supercapacitor electrode presented the power density of 6398.5 W/kg with an energy density of 344.8 Wh/kg under the current rate value of 40 mA (current density of 6.5 A·g^−1^).

**Figure 5:**
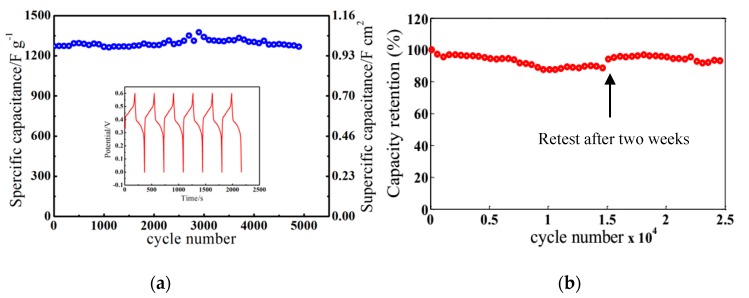
(**a**) Cycling performance of the MnO_2_–MWNT–Ni foam electrode. (**b**) Capacity retention property of the MnO_2_–MWNT–Ni foam electrode.

The cycle-life performance was tested within a potential window of 0 to 0.60 V. As shown in Figure 5a, a high specific capacitance of 1350.42 F·g^−1^ was reached after initial activation cycles, and the specific capacitance decreased to 1267.84 F·g^−1^ following 4600 cycles, exhibiting 93.9% retention. In a long cycle-life test, the electrode showed an excellent rate capability with 92.5% retention after 25,000 cycles (Figure 5b). Interestingly, the capacitance increased by 3% after retesting two weeks later. A MnO_2_–MWNT–Ni foam electrode with the addition of CNT powder presented a good electrochemical performance with 1.0 F·cm^−2^ areal capacitance and 77% retention after 3000 cycles [22]. A MnO_x_–CNT–Ni foam electrode with CNTs grown on an Fe catalyst film showed a specific capacitance of 462 F·g^−1^ [23]. A MnO_2_–CNT–Ni mesh electrode displayed a specific capacitance of 1072 F·g^−1^ [30]. Compared with the above CNT growth and addition techniques, the direct growth of MWNTs demonstrates advantages in supercapacitor developments for the reduction of the charge transfer resistance due to better MWNT–substrate contacts and long cycle life from strong MWNT adhesion. For traditional MWNT-based electrodes from CVD growth, an intermittent catalyst layer is required to grow nanotubes. Nanotubes grow upon this catalyst layer, resulting in high contact resistance and less binding strength between the MWNTs and the current collector. Meanwhile, the in situ deposition of MnO_2_ on MWNTs helps to stabilize the mesoporous structures over long test cycles, resulting in long-term stability.

## 4. Conclusions

We have successfully prepared MnO_2_–MWNT–Ni foam supercapacitor electrodes via hydrothermal MnO_2_ synthesis after growing MWNTs directly on Ni foam. The composite electrode exhibited both double-layer capacitance and faradic pseudocapacitance properties. The charge transfer resistance of the MnO_2_–MWNT–Ni foam electrode dropped to 0.046 Ω from 0.411 Ω for the MnO_2_–Ni foam electrode. In the three-electrode setup, the MnO_2_–MWNT–Ni foam electrode demonstrated excellent electrochemical properties with the specific capacitance of 1350.42 F·g^−1^ at the current density of 6.5 A·g^−1^ (40 mA rate value) and a high capacitance retention of 92.5% after 25,000 cycles. A power density of 6398.5 W·kg^−1^ was reached with an energy density of 344.8 Wh·kg^−1^. Direct MWNT growth shows great advantages for the supercapacitor application, as it results in a low charge transfer resistance, a reduction of the contact resistance, and strong MWNT adhesion with the current collector.

## Supplementary Materials

The following is available online at https://www.mdpi.com/2079-4991/9/5/703/s1, Figure S1: Comparisons of cyclic voltammetry (CV) properties for different electrodes.
