# Supplementary material for: Directly Grown Multiwall Carbon Nanotube and Hydrothermal MnO2 Composite for High-Performance Supercapacitor Electrodes
Source: Nanomaterials (Basel). 2019 May 6;9(5):703. doi: 10.3390/nano9050703 (PMC6566365; doi:10.3390/nano9050703)
Supplement: Supplementary file 1 [file nanomaterials-09-00703-s001.pdf]

Electronic Supplementary Information

Direct grown multiwall carbon nanotube and hydrothermal MnO_2 composite for high-performance supercapacitor electrodes

Li Li †, Lihui Chen †, Weijin Qian, Fei Xie and Changkun Dong *

Institute of Micro-nano Structures & Optoelectronics, Wenzhou University, Wenzhou 325035, Zhejiang, China; lili18767702665@gmail.com (L.L.); chenlihui19880416@gmail.com (L.C.); weijinqian@wzu.edu.cn (W.Q.); xiefei600@gmail.com (F.X.)

* Correspondence: dck@wzu.edu.cn; Tel: +86-577-86689067

† These authors contributed equally to this work.

Index

SI-1 Comparisons of cyclic voltammetry (CV) properties for different electrodes

Reference

SI-1 Comparisons of CV properties for different electrodes

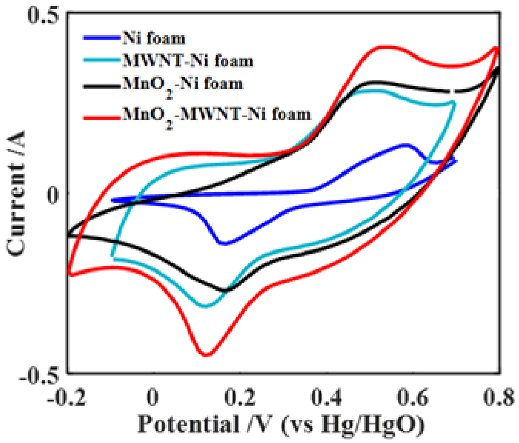

Figure S1. Cyclic voltammetry (CV) curves of four types of electrodes tested in 6 M KOH electrolyte.

CV curves of four types of electrodes at a scan rate of 100 mV/s are shown in Figure S1, showing the faradic pseudocapacitance of NiO and the reversible properties of the composite electrodes. The appearance of pseudocapacitance behavior at different potential ranges contributed to the different CV shapes in Figure 4 and Figure S1 [1].

Reference

1. Tian, Y.; Liu, Z. Y.; Xue, R.; Huang, L. P. An efficient supercapacitor of three-dimensional MnO_2 film prepared by chemical bath method, *J. Alloy. Compd.* 2016, *671*, 312-317.
